# *Cryptosporidium* in Rabbits: A Global Systematic Review and Meta‐Analysis of Prevalence, Species/Genotypes Distribution and Zoonotic Significance

**DOI:** 10.1002/vms3.70309

**Published:** 2025-03-19

**Authors:** Ali Ghorbani, Ali Asghari, Mohammad Reza Mohammadi, Milad Badri, Laya Shamsi, Fatemeh Hanifeh, Behnam Mohammadi‐Ghalehbin, Saiyad Bastaminejad

**Affiliations:** ^1^ Department of Microbiology and Virology, School of Medicine Kerman University of Medical Sciences Kerman Iran; ^2^ Medical Microbiology Research Center Qazvin University of Medical Sciences Qazvin Iran; ^3^ Department of Bacteriology, Faculty of Medical Sciences Tarbiat Modares University Tehran Iran; ^4^ Department of Pathobiology, Faculty of Veterinary Medicine Urmia University Urmia Iran; ^5^ Department of Biology, Faculty of Science Danesh Alborz University Abyek Iran; ^6^ Zoonoses Research Center Ardabil University of Medical Sciences Ardabil Iran; ^7^ Department of Genetics and Molecular Medicine, School of ParaMedicine Ilam University of Medical Sciences Ilam Iran

**Keywords:** *Cryptosporidium* spp, prevalence, rabbits, species, subtypes, systematic review

## Abstract

**Background:**

This systematic review and meta‐analysis assessed the global prevalence, species/genotype distribution and zoonotic impact of *Cryptosporidium* in rabbits.

**Methods:**

A systematic search of PubMed, Scopus and Web of Science was performed for studies from 2000 to 25 October 2024 on *Cryptosporidium* spp. in rabbits. Data on publication/implementation years, prevalence rates, rabbit types, diagnostics, countries and species/genotypes were collected. A meta‐analysis with random‐effects models estimated overall prevalence and assessed heterogeneity using the *I* ^2^ index. A sensitivity analysis evaluated the robustness of the results.

**Results:**

This systematic review included 26 studies with 6093 rabbits from 9 countries, revealing a pooled *Cryptosporidium* spp. prevalence of 9% (95% CI: 6%–13.4%). Three zoonotic species were found in rabbits: *Cryptosporidium cuniculus* in 18 studies and each of *C. parvum* and *C. andersoni* in 1 study. The isolates included 2 genotypes of *C. cuniculus* (Va, Vb) and 1 genotype of *C. parvum* (IIc), along with 18 subtypes of *C. cuniculus* (VaA16, VaA18, VaA31, VbA18, VbA19, VbA21, VbA22, VbA23, VbA24, VbA25, VbA26, VbA28, VbA29, VbA31, VbA32, VbA33, VbA35 and VbA36). Among these, 11 subtypes (VbA19, VbA22–VbA26, VbA28, VbA29 and VbA31–VbA33) are identified as zoonotic. Pet rabbits had the highest *Cryptosporidium* spp. pooled prevalence at 21.9% (95% CI: 14.7%–31.3%), followed by farmed rabbits at 9.7% (95% CI: 5.1%–17.8%), wild rabbits at 8.8% (95% CI: 4.8%–15.5%) and laboratory rabbits at 1% (95% CI: 0.3%–3.1%), with higher rates noted in Africa and the AFR WHO region.

**Conclusions:**

This study assessed the global distribution of *Cryptosporidium* spp. in rabbits, highlighting its zoonotic implications. It serves as a key resource for researchers, veterinarians and public health officials for future studies and control strategies.

## Graphical abstract

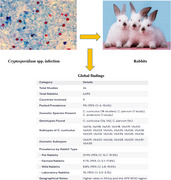

## 1 Introduction

*Cryptosporidium* is a protozoan parasite that causes cryptosporidiosis, leading to millions of diarrhoea cases annually, 12.9 million disability‐adjusted life‐years (DALYs), and approximately 48,000 deaths globally (Khalil et al. 2018). Notably, it ranks as the sixth most common cause of diarrhoeal death in children under five worldwide and is linked to long‐term consequences like stunted growth and malnutrition (Tipu et al. 2024). The faecal‐oral pathway is the main method of transmission, which can occur by hand‐to‐mouth contact or by consuming food and water tainted with *Cryptosporidium* spp. oocysts (Putignani and Menichella 2010).

Among the 44 recognized *Cryptosporidium* species and over 120 genotypes, 19 species and 4 genotypes were reported in humans, with *C. hominis*, *C. parvum*, *C. meleagridis*, *C. canis* and *C. felis* being the most common. Overall, *C. hominis*, *C. parvum*, *C. meleagridis*, *C. canis*, *C. felis*, *C. ubiquitum*, *C. cuniculus*, *C. viatorum*, *C. muris*, *C. andersoni*, *C. erinacei*, *C. tyzzeri*, *C. bovis*, *C. suis*, *C. scrofarum*, *C. occultus*, *C. xiaoi*, *C. fayeri*, *C. ditrichi*, *C. mortiferum*, mink genotype, skunk genotype and horse genotype have been reported in humans (U. Ryan et al. 2021; U.M. Ryan et al.; Yang et al. 2021). *C. hominis* and *C. parvum* account for approximately 95% of human infections, with *C. meleagridis*, *C. felis*, *C. canis* and *C. ubiquitum* following. Certain species, including *C. meleagridis*, *C. canis*, *C. felis*, *C. viatorum* and *C. muris*, are more frequently found in humans in developing countries, whereas others like *C. ubiquitum*, *C. cuniculus* and *C. mortiferum* are mainly present in developed countries (Chalmers et al. 2019; U. Ryan et al. 2021; Yang et al. 2021).

Because there are currently no highly effective medications to prevent or control cryptosporidiosis, the identification and genetic characterization of *Cryptosporidium* spp. from animals and the environment are essential for determining the sources of infection, modes of transmission and possible human infection risk (Cacciò and Pozio 2006; Jumani et al. 2021). Rabbits, often kept as pets, livestock and laboratory animals, can significantly influence the epidemiology and transmission of *Cryptosporidium* spp. to humans (Mullan and Main 2006; González‐Redondo and Contreras‐Chacón 2012; Egan et al. 2024). Thus, the purpose of this study was to examine the distribution and prevalence of *Cryptosporidium* species, genotypes and subtypes in rabbits by gathering and analysing the body of knowledge in this area through a meta‐analysis and systematic review.

## 2 Methods

### 2.1 Study Reporting and Registering

The Preferred Reporting Items for Systematic Reviews and Meta‐Analyses (PRISMA) criteria were followed in the conduct of this systematic review and meta‐analysis (Moher et al. 2015; Page et al. 2021). This systematic review and meta‐analysis protocol is registered on the Open Science Framework (OSF) with the DOI https://doi.org/10.17605/OSF.IO/2ZG3Y.

### 2.2 Research Questions

The following were the main research questions this systematic review addressed: How common are *Cryptosporidium* spp. in rabbit populations around the world? Which *Cryptosporidium* species/genotypes were isolated from rabbits? Do isolates of *Cryptosporidium* spp. from rabbits have any possible zoonotic implications?

### 2.3 Search Strategy

Two independent reviewers conducted systematic searches of the electronic databases Web of Science, PubMed and Scopus using the search phrases ‘*Cryptosporidium*’, ‘cryptosporidiosis’, ‘rabbits’, ‘epidemiology’, ‘genetic diversity’, ‘genotype’, ‘species’ and ‘zoonotic potential’. The keywords were effectively combined with the Boolean operators AND and OR. Additionally, Google Scholar and references from related articles were used to verify the number of articles identified in the systematic search. After importing the data, EndNote X7 removed duplicate articles, and two researchers independently evaluated the remaining studies.

### 2.4 Eligibility Criteria

This study is a global systematic review and meta‐analysis conducted without language or geographical restrictions, covering the period from 2000 to 25 October 2024. All cross‐sectional studies with available full text or extractable epidemiological details that specified both total and infected rabbits and documented the prevalence of *Cryptosporidium* spp. in these animals using genetic, serological or microscopic methods were included in this systematic review. Including some studies with small sample sizes supports our goal of providing a comprehensive analysis and ensures our findings remain generalizable. Some studies that did not mention the total sample size but mentioned the positive cases were included only to complete the information on the distribution of species and genotypes. Reviews, case studies, letters to the editor, commentaries, animal research unrelated to rabbits, human studies, experimental reports, studies with low‐quality assessment scores and studies with unavailable full text or unextractable epidemiological information were among the exclusion criteria.

### 2.5 Study Selection

The titles and abstracts of all identified studies were reviewed for eligibility by two independent reviewers. Other authors then evaluated the entire texts of studies that might be qualified. When necessary, a lead reviewer (A.A.) was consulted in order to resolve disagreements through conversation.

### 2.6 Data Extraction and Management

A pre‐made form that recorded study details (authors, year of publication, location), sample size, rabbit type (pet, farmed, laboratory and wild), prevalence rates of *Cryptosporidium* spp., isolated species/genotypes and diagnostic techniques was used to extract the data. Disagreements were settled by consensus after two reviewers independently finished the data extraction.

### 2.7 Quality Assessment

Key criteria, including sample size, participant descriptions, data analyses, trustworthy objectives, suitable statistical methods, confounding factors and subgroups, were covered by the Joanna Briggs Institute (JBI) checklist for reporting prevalence data in systematic reviews and meta‐analyses (Munn et al. 2014). Articles with scores ≤3 were not included; those with scores between 4 and 7 were categorized as medium quality, and those with scores above 7 as high qualities.

### 2.8 Software and Statistical Analysis

Comprehensive Meta‐Analysis (CMA) v3 software was used to conduct statistical analyses. Throughout the analysis, a *p* value of less than 0.05 was deemed statistically significant. A random‐effects model was used to pool the prevalence data in order to take study heterogeneity into consideration. The publication bias was investigated using a funnel plot and Egger's regression test. The variability was measured using the *I* ^2^ statistic; 25%, 50% and 75% *I* ^2^ values were considered low, moderate and high heterogeneity, respectively. Subgroup analyses were conducted according to sample size, WHO regions, nation, continent, diagnostics and year of publication. A descriptive analysis of *Cryptosporidium* spp. genetic diversity summarized the various species/genotypes and their possible zoonotic transmission implications.

### 2.9 Sensitivity Analysis

A sensitivity analysis was conducted to evaluate the robustness of the results. The meta‐analysis was re‐examined, with individual datasets and studies removed, and the effect on overall prevalence estimates was evaluated.

## 3 Results

### 3.1 Number of Included Studies

**FIGURE 1:**
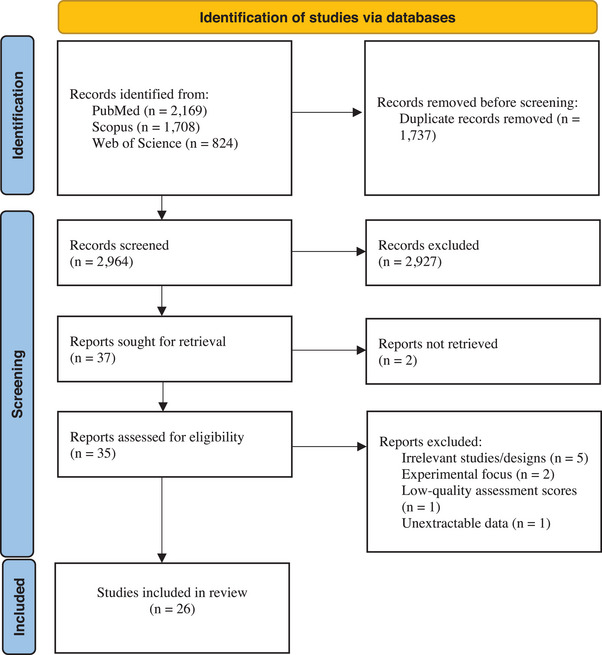
The PRISMA 2020 flow diagram depicting the process of included studies in the present review.

The systematic search of this study found 4701 articles across electronic databases: 2169 in PubMed, 1708 in Scopus and 824 in Web of Science. After removing duplicates, 2964 articles remained. Following the initial screening of titles and abstracts, 35 articles were deemed potentially eligible. Full‐text and quality assessments were conducted for these studies, leading to the inclusion of 26 studies that met the eligibility criteria (Tian et al. 2002; Shiibashi et al. 2006; Soltane et al. 2007; Ni et al. 2008; Men JingTao et al. 2009; Chalmers et al. 2009; Nolan et al. 2010, 2013; Shi et al. 2010; Zhang et al. 2012, 2018; Liu et al. 2014; Akinkuotu et al. 2016; Yang et al. 2016; Zahedi et al. 2016, 2018; Heker et al. 2016; Koehler et al. 2016; Marhoon et al. 2018; Elshahawy and Elgoniemy 2018; Ayinmode and Agbajelola 2019; Al‐Dahhan and Zghair 2020; Naguib et al. 2021; Baz‐González et al. 2022; Lu et al. 2022; Rego et al. 2023). Study exclusions occurred due to irrelevant studies/designs (*n* = 5), experimental focus (*n* = 2), low‐quality assessment scores (*n* = 1) and unextractable data (*n* = 1) (Figure 1).

### 3.2 Study Characteristics

The studies encompassed varied geographic locations, representing nine countries across five continents: Africa, Asia, Europe, Oceania and South America; and five WHO regions: AFR, AMR, EMR, EUR and WPR. Included studies were published between 2002 and 2023, with sample sizes ranging from 27 to 1081 rabbits and a total of 6093 rabbits assessed across all studies. The diagnostic techniques used included genetic methods (20 studies), serological tests (1 study) and microscopic examinations (5 studies) (Tables 1 and 2).

**TABLE 1:** Key characteristics of 26 articles on the prevalence and distribution of *Cryptosporidium* species/genotypes in rabbits.

Author, year	Host type	Time tested	Country	Total no.	Infected no.	Prevalence (%)	Method	Species (no.)	Genotypes/subtypes (no.)
Tian (2002)	Farmed rabbits	UC^a^	China	35	1	2.8	MOL^b^	*C. cuniculus* (1)	—
Shiibashi (2006)	Pet rabbits	2002	Japan	96	21	21.9	MIC^c^	*Cryptosporidium* spp. (21)	—
Soltane (2007)	Farmed rabbits	2003–2004	Tunisia	178	0	0	MOL	—	—
Ni (2008)	Farmed rabbits	UC	China	69	11	15.9	MIC	*Cryptosporidium* spp. (11)	—
Men (2009)	Farmed rabbits	UC	China	287	97	33.8	MOL	*C. cuniculus* (97)	—
Chalmers (2009)	Wild rabbits	2008	United Kingdom	—	1^f^	—	MOL	*C. cuniculus* (1)	VaA18 (1)
Shi (2010)	Farmed rabbits	2007–2008	China	1081	37	3.4	MOL	*C. cuniculus* (37)	VbA29 (18), VbA35 (4) and VbA36 (8)
Nolan (2010)	Wild rabbits	UC	Australia	176	12	6.8	MOL	*C. cuniculus* (12)	VbA23R3 (11) and VbA26R4 (1)
Zhang (2012)	Farmed rabbits	2008–2010	China	378	9	2.4	MOL	*C. cuniculus* (9)	VbA21 (6) and VbA32 (3)
Nolan (2013)	Wild rabbits	2009–2011	Australia	263	22	8.3	MOL	*C. cuniculus* (22)	VbA22R4, VbA23R3, VbA24R3, VbA25R4 and VbA26R4
Liu (2014)	Laboratory rabbits	UC	China	290	3	1	MOL	*C. cuniculus* (3)	VaA31 (3)
Akinkuotu (2016)	Farmed rabbits	2014	Nigeria	27	25	92.6	SER^d^	*Cryptosporidium* spp. (25)	—
Zahedi (2016)	Wild rabbits	2013–2015	Australia	106	14	13.2	MOL	*C. cuniculus* (14)	VbA23 (9) and ND^e^ (5)
Yang (2016)	Farmed rabbits	2015–2016	China	215	24	11.2	MOL	*C. cuniculus* (24)	VbA28 (2), VbA29 (16) and VbA32 (3)
Koehler (2016)	Wild rabbits	2011–2015	Australia	321	7	2.2	MOL	*C. cuniculus* (7)	VbA25 (2), VbA26 (4) and VbA24 (1)
Heker (2016)	Farmed rabbits	2012	Brazil	55	7	12.7	MOL	*C. cuniculus* (7)	VbA21 (7)
Elshahawy and Elgoniemy (2018)	Farmed rabbits	2011–2012	Egypt	298	45	15.1	MIC	*Cryptosporidium* spp. (45)	—
Marhoon (2018)	Wild rabbits	2016–2017	Iraq	55	21	38.2	MIC	*Cryptosporidium* spp. (21)	—
Zahedi (2018)	Wild rabbits	2013–2015	Australia	672	96	14.3	MOL	*C. cuniculus* (96)	VbA18 (12), VbA23 (46), VbA25 (16), VbA26 (8), VbA28 (2), VbA29 (5) and ND (7)
Zhang (2018)	Farmed rabbits	2015–2017	China	321	11	3.4	MOL	*C. cuniculus* (11)	VbA24 (5) and ND (6)
Ayinmode and Agbajelola (2019)	Farmed rabbits	2016	Nigeria	107	4	3.7	MOL	*C. parvum* (4)	IIc (4)
Al‐Dahhan and Zghair (2020)	Wild rabbits	2019	Iraq	180	47	26.1	MIC	*Cryptosporidium* spp. (47)	—
Naguib (2021)	Farmed rabbits	2015–2016	Egypt	235	28	11.9	MOL	*C. cuniculus* (28)	VbA19 (1), VbA33 (15) and ND (12)
Baz‐González (2022)	Wild rabbits	2015–2017	Spain	100	4	4	MOL	*C. cuniculus* (1), ND (3)	VbA26R3 (1) and ND (3)
Lu (2022)	Farmed rabbits	UC	China	—	6^f^	—	MOL	*C. cuniculus* (6)	VbA24 (1), VbA29 (2), VbA31 (2) and VbA33 (1)
Rego (2023)	Wild rabbits	2012–2021	Spain	550	7	1.3	MOL	*C. cuniculus* (6), *C. andersoni* (1)	VaA16 (1), VaA18 (2), VbA24 (1), VbA31 (1) and ND (1)

^a^ Unclear.

^b^ Molecular detection.

^c^ Microscopic detection.

^d^ Serological detection.

^e^ Not determined.

^f^ These studies, mentioning only positive cases, were included solely to enhance the information on *Cryptosporidium* spp. infection in rabbits and were excluded from statistical analyses.

**TABLE 2:** Subgroup analysis of *Cryptosporidium* spp. prevalence in rabbits based on publication years, continents, WHO regions, countries, sample sizes and diagnostic methods.

Subgroup variable	Prevalence % (95% CI)	Heterogeneity (*Q*)	No. studies	df (*Q*)	*I* ^2^ (%)	*p* value
Publication year						
≤2014	6 (2.5–13.5)	248.9	10	9	96.4	*p* < 0.05
>2014	11.1 (7–17.1)	192.5	14	13	93.2	*p* < 0.05
Continent						
Africa	14.6 (5.3–34.2)	52.2	5	4	92.3	*p* < 0.05
Asia	9.5 (4.6–18.5)	296	11	10	96.6	*p* < 0.05
Europe	2.2 (0.7–6.4)	3.3	2	1	69.7	*p* < 0.05
Oceania	8 (4.6–13.6)	32.9	5	4	87.9	*p* < 0.05
South America	12.7 (6.2–24.3)	0	1	0	0	*p* < 0.05
WHO region						
AFR	40.4 (0.2–99.5)	41.7	2	1	97.6	*p* < 0.05
AMR	12.7 (6.2–24.3)	0	1	0	0	*p* < 0.05
EMR	17.7 (10–29.3)	38.2	5	4	89.5	*p* < 0.05
EUR	2.2 (0.7–6.4)	3.3	2	1	69.7	*p* < 0.05
WPR	7.3 (4.2–12.2)	287.4	14	13	95.5	*p* < 0.05
Country						
Australia	8 (4.6–13.6)	32.9	5	4	87.8	*p* < 0.05
Brazil	12.7 (6.2–24.3)	0	1	0	0	*p* < 0.05
China	5.7 (2.1–14.7)	245.2	8	7	97.1	*p* < 0.05
Egypt	13.7 (10.9–17.2)	1.1	2	1	11.8	*p* < 0.05
Iraq	30.9 (20.7–43.5)	2.9	2	1	66.2	*p* < 0.05
Japan	21.9 (14.7–31.3)	0	1	0	0	*p* < 0.05
Nigeria	40.4 (0.2–99.5)	41.7	2	1	97.6	*p* < 0.05
Spain	2.2 (0.7–6.4)	3.3	2	1	69.7	*p* < 0.05
Tunisia	0.3 (0–4.3)	0	1	0	0	*p* < 0.05
Sample size						
≤100	20.8 (9.5–39.9)	57.7	7	6	89.6	*p* < 0.05
>100	6.5 (4–10.3)	361.8	17	16	95.6	*p* < 0.05
Diagnostics						
MIC	22.4 (15.8–30.7)	19.2	5	4	79.2	*p* < 0.05
MOL	5.4 (3.3–8.9)	330.4	18	17	94.8	*p* < 0.05
SER	92.6 (74.8–98.1)	0	1	0	0	*p* < 0.05

### 3.3 Pooled Prevalence of *Cryptosporidium* spp. In Rabbits

**FIGURE 2:**
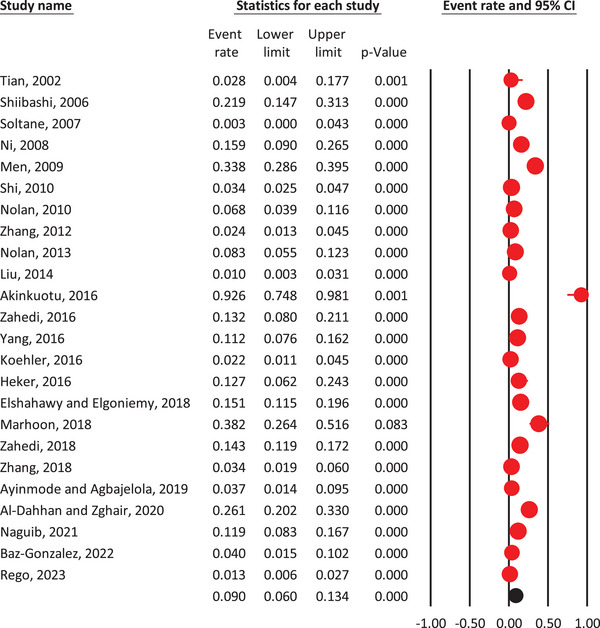
The pooled prevalence of *Cryptosporidium* spp. in rabbits, based on data from 24 studies, using a random‐effects model and 95% confidence intervals.

The weighted prevalence of *Cryptosporidium* spp. in rabbit populations was 9% (95% CI: 6%–13.4%) according to the random‐effects model (Figure 2). Significant heterogeneity was observed among studies (*I* ^2^ = 94.8%, *p* < 0.001), indicating variability in prevalence estimates. Subgroup analyses showed significant variation in prevalence based on studied variables (Table 2 and Figures S1–S6).

### 3.4 Pooled Prevalence of *Cryptosporidium* spp. In Rabbits by Animal Type

**FIGURE 3:**
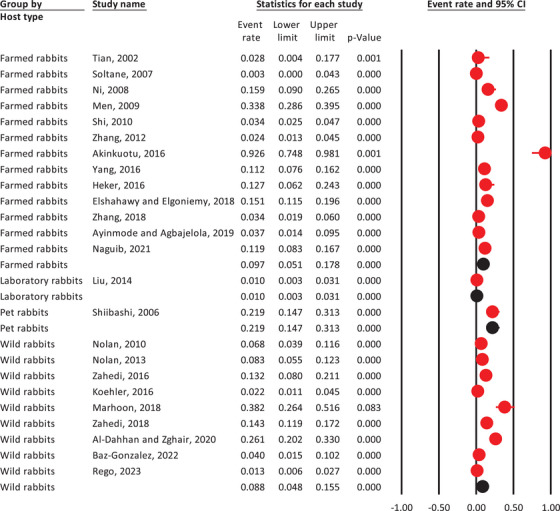
The weighted prevalence of *Cryptosporidium* spp. in rabbits, based on host type, using a random‐effects model and 95% confidence intervals.

Pet rabbits had the highest *Cryptosporidium* spp. pooled prevalence at 21.9% (95% CI: 14.7%–31.3%), followed by farmed rabbits at 9.7% (95% CI: 5.1%–17.8%), wild rabbits at 8.8% (95% CI: 4.8%–15.5%) and laboratory rabbits at 1% (95% CI: 0.3%–3.1%) (Figure 3).

### 3.5 Species Distribution of *Cryptosporidium* spp. In Rabbits

Three zoonotic species were found in rabbits: *C. cuniculus* in 18 studies and each of *C. parvum* and *C. andersoni* in 1 study (Table 1).

### 3.6 Genotype and Subtype Distribution of *C. parvum* and *C. cuniculus* in Rabbits

The *Cryptosporidium* isolates comprised 2 genotypes of *C. cuniculus* (Va, Vb) and 1 genotype of *C. parvum* (IIc), along with 18 subtypes of *C. cuniculus* (VaA16, VaA18, VaA31, VbA18, VbA19, VbA21, VbA22, VbA23, VbA24, VbA25, VbA26, VbA28, VbA29, VbA31, VbA32, VbA33, VbA35 and VbA36). Among these, 11 subtypes (VbA19, VbA22–VbA26, VbA28, VbA29 and VbA31–VbA33) were identified as zoonotic (Table 1).

### 3.7 Quality Evaluation

The quality assessment of included studies using the JBI checklist indicated that 10 studies were of high quality (scores >7) and 16 were of medium quality (scores between 4 and 7) (Table S1).

### 3.8 Sensitivity Analysis

The sensitivity analysis demonstrated that the overall prevalence estimate remained stable, with a slight range of variation from 11.8% to 13.5% when individual studies were excluded (Figure S7).

### 3.9 Publication Bias

**FIGURE 4:**
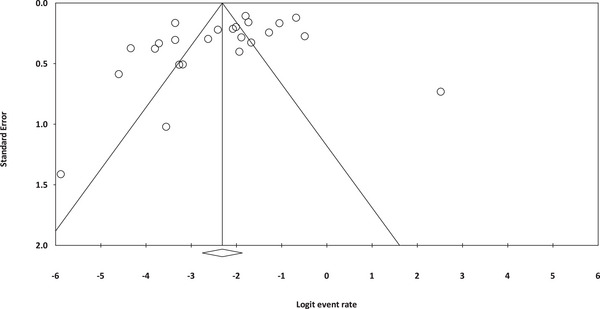
The funnel plot illustrates the publication bias within this study.

Assessment for publication bias using Egger's regression indicated no significant bias (intercept = −3.481, *p* = 0.06) (Figure 4).

## 4 Discussion

Rabbits are commonly kept as pets, livestock and laboratory animals, and they can significantly impact the spread of *Cryptosporidium* spp. to humans and other domestic animals (Mullan and Main 2006; González‐Redondo and Contreras‐Chacón 2012; Egan et al. 2024). These parasites are well‐known for causing gastrointestinal infections, which can result in severe diarrhoea and other health issues (Gerace et al. 2019). The detection of *Cryptosporidium* spp. in rabbit populations has raised concerns among veterinarians and public health officials, as rabbits can serve as reservoirs for the pathogen (Egan et al. 2024). Moreover, the rising popularity of rabbits as companion animals has increased their contact with humans, inadvertently raising the risk of zoonotic transmission. Hence, this study aimed to analyse the distribution and prevalence of *Cryptosporidium* species/genotypes in rabbits through a meta‐analysis and systematic review of existing knowledge.

Given the pathogenic significance of *Cryptosporidium* spp. in animals and its potential transmission to vulnerable populations (immunocompromised individuals, children and the elderly) (Hunter and Nichols 2002; Dong et al. 2020), various systematic reviews and meta‐analyses have reported infection rates for different animals: camels (13.8%, 95% CI: 10.3%–18.4%) (Mahdavi et al. 2024), pigs (16.3%, 95% CI: 15%–17.6%) (Chen et al. 2023), cats (6%, 95% CI: 4%–8%) (Taghipour et al. 2021), *Equus* species (7.6%, 95% CI: 4.8%–10.8%) (Li et al. 2022), rodents (17%, 95% CI: 13%–20%) (Taghipour et al. 2020) and dogs (6%, 95% CI: 4%–9%) (Taghipour et al. 2020). Despite variations in the number of studies, geographical areas, diagnostic methods and sample sizes, the present study found a pooled prevalence of 9% (95% CI: 6%–13.4%) for *Cryptosporidium* spp. in rabbits, which was higher than that found in cats, *Equus* animals and dogs. This suggests that the role of rabbits in transmitting *Cryptosporidium* spp. infections to humans should not be underestimated. Moreover, the interaction between rabbits and other animals, such as pets and livestock, raises concerns about cross‐species transmission. Hence, understanding the ecological dynamics that facilitate the spread of *Cryptosporidium* spp. is essential. To mitigate transmission risk, proper husbandry practices and regular health assessments should be implemented. The sensitivity analysis showed that the overall prevalence estimate was stable, varying slightly from 11.8% to 13.5% when individual studies were excluded.

Analysis of the molecular data revealed 3 zoonotic species present in rabbits: *C. cuniculus* in 18 studies and each of *C. parvum* and *C. andersoni* in 1 study. The presence of these species underscores the public health risks of zoonotic transmission from rabbits to humans. The high prevalence of *C. cuniculus* in rabbits confirms them as the primary host and reservoir for this particular zoonotic species. In contrast, the lower prevalence of *C. parvum* and *C. andersoni* indicates that their contribution to the overall zoonotic risk from rodents is relatively minor. Rabbits appear to have contracted *C. parvum* and *C. andersoni* from environmental, animal and/or human sources. Besides being at risk to their health, they may significantly contribute to the transmission of these zoonotic species. Overall, the known hosts for *C. cuniculus* include humans, rabbits, Eastern grey kangaroos (*Macropus giganteus*) and alpacas (*Vicugna pacos*). It has also been detected in wastewater treatment plants, rivers and water sources (Hu et al. 2014; Koehler et al. 2018, 2014; Puleston et al. 2014; Swaffer et al. 2018). *C. parvum* infects a diverse range of hosts, including ungulates, wildlife (such as carnivores, rodents, non‐human primates, marine mammals and fish), and is the primary zoonotic species affecting humans, especially in rural areas with regular livestock contact (Zahedi and Ryan 2020; U. Ryan et al. 2021; Yang et al. 2021). *C. andersoni* is frequently found in humans, cattle, sheep, goats, deer and rodents (Lindsay et al. 2000; Huang et al. 2018; Guo et al. 2021; J. Chen et al. 2021; S. Chen et al. 2021).

Pet rabbits showed the highest pooled prevalence of *Cryptosporidium* spp. at 21.9% (95% CI: 14.7%–31.3%), followed by farmed rabbits at 9.7% (95% CI: 5.1%–17.8%), wild rabbits at 8.8% (95% CI: 4.8%–15.5%) and laboratory rabbits at 1% (95% CI: 0.3%–3.1%). This variation in prevalence rates highlights the potential risk factors associated with different housing and management conditions. Pet rabbits, often kept in close proximity to human environments, may have higher exposure to sources of infection compared to their farmed and wild counterparts. Farmed rabbits, while more controlled in their environment, can still encounter *Cryptosporidium* spp. through suboptimal hygiene practices or contaminated feed and water sources. Wild rabbits, exposed to natural habitats where they interact with diverse wildlife, may also face a unique set of risks that contribute to their infection rates. The much lower prevalence in laboratory rabbits likely reflects the more stringent biosecurity measures and health monitoring protocols typically implemented in research settings. Of note, some analyses in this study rely on limited information or single dataset/study, which may mislead readers. Therefore, caution is advised when interpreting the results.

Most cases of *C. cuniculus* in humans and rabbits are attributed to subtype family Vb (Koehler et al. 2014; Garcia‐R et al. 2017; Lebbad et al. 2021; U. Ryan et al. 2021). In humans, the reported subtypes include VaA11, VaA18, VaA19–VaA22, VaA23, VbA13, VbA15, VbA17, VbA20, VbA22, VbA23, VbA24–VbA34, VbA36, VbA37 and VbA38 (Robinson et al. 2010; Chalmers et al. 2011; Hadfield and Chalmers 2012; Garcia‐R et al. 2020; Guy et al. 2021; Lebbad et al. 2021; U. Ryan et al. 2021). This review identified 18 subtypes in rabbits (VaA16, VaA18, VaA31, VbA18, VbA19, VbA21, VbA22, VbA23, VbA24, VbA25, VbA26, VbA28, VbA29, VbA31, VbA32, VbA33, VbA35 and VbA36). Among these, 11 subtypes (VbA19, VbA22‐VbA26, VbA28, VbA29 and VbA31–VbA33) were identified as zoonotic (U. Ryan et al. 2021). Understanding the zoonotic potential of these subtypes is critical for public health and veterinary practices. Researchers have emphasized the importance of surveillance in both human and rabbit populations to monitor the emergence and spread of these subtypes. Furthermore, the intersection of human and rabbit habitats increases the risk of transmission, necessitating awareness among pet owners and those working in agricultural settings. The three main *C. parvum* gp60 genotype/subtype families in humans are IIa, IId and IIc, with IIc primarily transmitted between humans, whereas IIa and IId are zoonotic (King et al. 2019; U.M. Ryan et al.; Yang et al. 2021). The analysis of data revealed that *C. parvum* genotype IIc was found in domestic rabbits in Nigeria (Ayinmode and Agbajelola 2019). Given the anthropogenic nature of this genotype and the insufficient information on animal infections, it suggests that the rabbits likely serve as mechanical carriers of *C. parvum* IIc from environmental sources or contaminated water and feed, rather than being truly infected or colonized. The presence of *C. parvum* genotype IIc in domestic rabbits underscores the potential risk of zoonotic transmission and highlights the need for further studies to understand the ecological dynamics of this pathogen. Rabbits, as mechanical carriers, might play a critical role in the transmission pathway of *C. parvum*, pointing to the significance of assessing water quality and feed sources in agricultural practices. Contaminated water supplies, often resulting from inadequate sanitation and waste management, could facilitate the spread of the parasite not only among animal populations but also to humans through direct or indirect contact. Additionally, the detection of this genotype in a domestic animal population emphasizes the interconnectedness of wildlife, livestock and human health, often referred to as the One Health concept. Further investigation into the environmental reservoirs of *C. parvum*, alongside improved biosecurity measures in rabbit farming, can mitigate potential outbreaks.

The analysis of publication years revealed that the pooled prevalence of *Cryptosporidium* spp. in rabbits was higher post‐2014 (11.1%, 95% CI: 7%–17.1%) than pre‐2014 (6%, 95% CI: 2.5%–13.5%). This shift in weighted prevalence indicates a possible increase in environmental exposure or changes in management practices affecting rabbit populations. Furthermore, the observed rise may correlate with broader ecological factors, such as alterations in land use, climate change or the emergence of novel parasite strains. Moreover, the weighted prevalence of *Cryptosporidium* spp. in Africa and South America was higher by 14.6% (95% CI: 5.3%–34.2%) and 12.7% (95% CI: 6.2%–24.3%), respectively, compared to other continents. Additionally, rabbits in the AFR and EMR WHO regions exhibited the highest infection rates, at 40.4% (95% CI: 0.2%–99.5%) and 17.7% (95% CI: 10%–29.3%), respectively. Notably, the highest infection rates were reported in Nigeria (40.4%), Iraq (30.9%), Japan (21.9%), Egypt (13.7%) and Brazil (12.7%). The significance of these findings underscores the need for targeted public health interventions in regions with elevated infection rates. The sample size‐based findings indicated that the pooled prevalence of *Cryptosporidium* spp. in rabbits was higher in samples below 100 (20.8%) compared to those above 100 (6.5%). This finding suggests that a larger sample size is necessary to accurately determine the true prevalence of an infection in a specific population and to better understand its current epidemiological situation. In the systematic review and meta‐analyses reporting prevalence data, it is preferable to report the overall prevalence based on diagnostic methods. In the present study, due to the lack of diversity in studies related to different diagnostic methods, we were unable to achieve a correct and comprehensive statistical analysis in this regard. However, in the present analyses, the highest and lowest pooled prevalence of *Cryptosporidium* spp. in rabbits were based on serological (92.6%) and molecular diagnostic methods (5.4%). Due to the limited information in this area, it is recommended to interpret the results of this section with caution.

The statistical analyses in this study showed no evidence of publication bias (*p* = 0.06). However, visual inspection of the funnel plot revealed some studies outside the funnel. Given that even slight variations can impact statistical outcomes, we must report the absence of publication bias based on these findings. In conclusion, the findings and interpretations of this study rely on the current information available, and future research could greatly influence these results. Additionally, because some analyses are based on individual studies, small sample sizes and unequal diagnostic methods, it is advisable to interpret the results with caution and acknowledge the limitations to avoid exaggeration and confusion.

## 5 Conclusion

Cryptosporidiosis can pose a significant challenge to the rabbit industry, affecting both animal welfare and economic viability. Moreover, the increasing popularity of rabbits as companionship animals has brought them into closer contact with humans, inadvertently enhancing the risk of zoonotic transmission. It is crucial for pet owners to maintain proper hygiene practices, such as regular cleaning of living spaces and careful handling of rabbits, to mitigate these risks. Overall, this review serves as a foundational resource for researchers, veterinarians and public health officials, providing critical insights for future studies and strategies aimed at controlling *Cryptosporidium* spp. infections in rabbits. By addressing the emerging zoonotic risks and monitoring prevalence trends, we can better protect both animal and human health in the face of ongoing environmental and ecological changes.

## Author Contributions

Ali Ghorbani, Ali Asghari and Behnam Mohammadi‐Ghalehbin planned and designed the study. Ali Asghari, Milad Badri, Mohammad Reza Mohammadi, Fatemeh Hanifeh and Laya Shamsi were involved in the methodology and data extraction. Ali Asghari conducted the statistical analysis. Ali Ghorbani, Ali Asghari, Saiyad Bastaminejad and Mohammad Reza Mohammadi wrote the manuscript and revised it. All authors have read and approved the final manuscript.

## Ethics Statement

The present study was approved by the Ethics Committee of Ardabil University of Medical Sciences, Ardabil, Iran (IR.ARUMS.REC.1403.427).

## Conflicts of Interest

The authors declare no conflicts of interest.

## Supporting information



Supporting Information

Supporting Information

Supporting Information

Supporting Information

Supporting Information

Supporting Information

Supporting Information

Supporting Information

## Data Availability

The datasets used and/or analysed during the current study are available in the online version.
