# Supplementary material for: Cryptosporidium in Rabbits: A Global Systematic Review and Meta‐Analysis of Prevalence, Species/Genotypes Distribution and Zoonotic Significance
Source: Vet Med Sci. 2025 Mar 19;11(2):e70309. doi: 10.1002/vms3.70309 (PMC11920741; doi:10.1002/vms3.70309)
Supplement: Supplementary file 1 — Supporting Information [file VMS3-11-e70309-s002.docx]

**Supplementary Table 1**

**JBI critical appraisal checklist applied for included studies**

| Author Name/Year | Sample was representative? | Participants appropriately recruited? | Sample size was adequate? | Study subjects and the setting described? | Data analysis conducted | Objective, standard criteria, reliably used? | Appropriate statistical analysis used | Confounding factors/ subgroups/ differences identified and accounted? | Subpopulations identified using objective criteria | Overall quality |
| --- | --- | --- | --- | --- | --- | --- | --- | --- | --- | --- |
| Tian, 2002 | Yes | Yes | Yes | Yes | Yes | Yes | No | No | No | 4/9 |
| Shiibashi, 2006 | Yes | No | Yes | Yes | No | Yes | No | No | No | 6/9 |
| Soltane, 2007 | Yes | No | Yes | No | Yes | No | Yes | No | No | 4/9 |
| Ni, 2008 | Yes | Yes | No | Yes | No | Yes | No | Yes | No | 5/9 |
| Men, 2009 | Yes | Yes | Yes | Yes | Yes | No | Yes | Yes | No | 7/9 |
| Chalmers, 2009 | Yes | Yes | Yes | Yes | Yes | No | Yes | No | No | 6/9 |
| Shi, 2010 | Yes | Yes | Yes | Yes | Yes | No | Yes | Yes | Yes | 8/9 |
| Nolan, 2010 | Yes | Yes | Yes | Yes | Yes | Yes | Yes | Yes | No | 8/9 |
| Zhang, 2012 | Yes | Yes | Yes | Yes | Yes | No | Yes | No | Yes | 7/9 |
| Nolan, 2013 | Yes | Yes | Yes | Yes | Yes | No | Yes | Yes | Yes | 8/9 |
| Liu, 2014 | Yes | No | Yes | Yes | Yes | Yes | Yes | No | Yes | 7/9 |
| Akinkuotu, 2016 | Yes | Yes | Yes | Yes | Yes | Yes | No | No | No | 4/9 |
| Zahedi, 2016 | Yes | No | Yes | Yes | No | Yes | No | No | No | 6/9 |
| Yang, 2016 | Yes | No | Yes | No | Yes | No | Yes | No | No | 4/9 |
| Koehler, 2016 | Yes | Yes | No | Yes | No | Yes | No | Yes | No | 5/9 |
| Heker, 2016 | Yes | Yes | Yes | Yes | Yes | No | Yes | Yes | No | 7/9 |
| Elshahawy and Elgoniemy, 2018 | Yes | Yes | Yes | Yes | Yes | No | Yes | No | No | 6/9 |
| Marhoon, 2018 | Yes | Yes | Yes | Yes | Yes | No | Yes | Yes | Yes | 8/9 |
| Zahedi, 2018 | Yes | Yes | Yes | Yes | Yes | Yes | No | No | No | 4/9 |
| Zhang, 2018 | Yes | No | Yes | Yes | No | Yes | No | No | No | 6/9 |
| Ayinmode and Agbajelola, 2019 | Yes | Yes | Yes | Yes | Yes | No | Yes | Yes | No | 7/9 |
| Al-Dahhan and Zghair, 2020 | Yes | Yes | No | Yes | No | Yes | No | Yes | No | 5/9 |
| Naguib, 2021 | Yes | Yes | Yes | Yes | Yes | Yes | No | No | No | 4/9 |
| Baz-Gonzalez, 2022 | Yes | No | Yes | Yes | No | Yes | No | No | No | 6/9 |
| Lu, 2022 | Yes | Yes | Yes | Yes | Yes | No | Yes | No | No | 7/9 |
| Rego, 2023 | Yes | Yes | No | Yes | No | Yes | No | Yes | No | 5/9 |
