# Supplementary material for: Cryptosporidium in Rabbits: A Global Systematic Review and Meta‐Analysis of Prevalence, Species/Genotypes Distribution and Zoonotic Significance
Source: Vet Med Sci. 2025 Mar 19;11(2):e70309. doi: 10.1002/vms3.70309 (PMC11920741; doi:10.1002/vms3.70309)
Supplement: Supplementary file 6 — Supporting Information [file VMS3-11-e70309-s007.docx]

**Supplementary Fig. 5.** The weighted prevalence of *Cryptosporidium* spp. in rabbits, based on sample size.
